# Connect Care Project, Bridging the Gap Between Acute and Post-Acute Care

**DOI:** 10.1007/s11606-025-09598-0

**Published:** 2025-05-14

**Authors:** Housam Hegazy, Koh-Eun Narm, Brian Pratt, Tiffany Bell, James Mangano, Tara Mathews, Sakshi Dutta, Christopher P Morley, Alyssa M Indelicato, Ilona Chepak, Harvir Singh Gambhir, Zachary Shepherd, Amy Tucker

**Affiliations:** 1 Division of Hospital Medicine, State University of New York Upstate Medical University, Syracuse, USA; 2 Department of Internal Medicine, State University of New York Upstate Medical University, Syracuse, USA; 3 Department of Public Health & Preventive Medicine, State University of New York Upstate Medical University, Syracuse, USA; 4 Emergency Department, State University of New York Upstate Medical University, Syracuse, USA; 5 Department of Family Medicine, State University of New York Upstate Medical University, Syracuse, USA; 6 Department of Psychiatry, State University of New York Upstate Medical University, Syracuse, USA; 7 Norton College of Medicine, State University of New York Upstate Medical University, Syracuse, USA; 8 Division of Cardiology, State University of New York Upstate Medical University, Syracuse, USA

## Abstract

**Background:**

Hospitals in the USA face increasing challenges with access and capacity, prompting strategies to optimize resources, enhance throughput, and improve patient care access.

**Objective:**

This study assesses the impact of an innovative clinic-based ambulatory service as an alternative to hospital-based outpatient care, including observation stays and emergency department (ED) follow-up.

**Setting and Participants:**

A retrospective review of observation-status medical admissions from March 2020 to April 2023 at SUNY Upstate University Hospital. Patients presented to the SUNY Upstate ED.

**Program Description:**

The Connect Care Project (CCP) introduced an ED-based hospitalist triage team and a hospitalist-led Connect Care (CC) clinic. The triage team identified ED patients needing expedited outpatient workup or close monitoring and follow up. Instead of hospital observation or ED follow up, these patients were referred to the CC clinic for needed workup, close monitoring, or follow-up.

**Program Evaluation and Results:**

Admissions under observation were compared 13 months before and after CCP implementation (November 2021). Chest pain, a common observation reason, was closely analyzed. Of 305,207 ED visits, observation admissions —especially for chest pain —significantly declined after CCP implementation.

**Discussion:**

The CCP model improved capacity, reduced cost, and streamlined patient flow. It is adaptable for broader implementation.

## INTRODUCTION

For years, constrained hospital inpatient capacity has been a challenge at many academic medical centers and has been exacerbated post-pandemic^1,2^. Among the contributing factors are increased length of stay (LOS) due to higher acuity patients, bottlenecks in post-acute care, such as limited availability of skilled nursing facility beds^2–5^, and over-use of emergency departments (ED) and inpatient encounters to address ambulatory-sensitive conditions^6–9^. The pandemic has also led to a nationwide decline in the number of staffed hospital beds, largely due to financial instability and workforce shortages^10^. As a result, many regions experience high hospital occupancy rates with fewer hospital beds per capita^10,11^. To address the critical need, hospitals have focused on improving patient throughput by increasing operational efficiency and on decreasing demand by shifting low acuity care to ambulatory settings. Initiatives that focus on driving more efficient throughput increase pressure on inpatient hospitalists, who are accountable for the largest share of inpatient volume^4,12,13^. Strategies to reduce demand include expanding ambulatory capacity for non-emergent conditions, reducing admissions by creating clinical pathways to allow safe ambulatory management for low-risk presentations of specific symptoms, and providing timely ambulatory follow-up for ED discharges to shift some of the hospital-based outpatient care to clinic-based care.

New York State has experienced a greater contraction in staffed hospital beds^10^and an overall higher average occupancy rate compared to the national average (79% versus 73%)^14^. Several regions in New York State, including central New York, routinely exceed the national benchmark for hospital occupancy, which is ideally close to 85%^11^. SUNY Upstate Medical University Hospital (Upstate) is an academic medical center with a Level I Trauma Center serving the upstate New York region. Occupancy in this center consistently exceeds 90%, and routinely reaches 100%^11^. Bed-occupancy rates exceeding 85% are strongly correlated with longer boarding times in emergency departments and longer LOS^15,16^.

When patients are hospitalized, they may be classified as either inpatient or outpatient levels of care. Inpatient care is reserved for conditions requiring hospital-based treatment due to severity or a longer expected duration of care. In contrast, outpatient care refers to services that do not necessitate hospital-based care. The observation level of care is a specific outpatient classification for hospitalized patients. It is a short-term hospital-based outpatient service designated for cases where outpatient medical evaluation or treatment can be completed in the hospital within a limited timeframe, or when the need for inpatient admission has not yet been determined. Common conditions warranting observation status include chest pain, syncope, dehydration, mild infections, or post-procedural monitoring^17^. Among these conditions, chest pain is a frequent reason for placing patients under observation. When a patient presents with chest pain, but the initial assessment rules out an acute, life-threatening condition—such as myocardial infarction—the patient may be admitted under observation status for further expedited outpatient evaluation. This approach allows clinicians to determine whether the symptoms require escalation to inpatient admission or if the patient can be safely discharged for outpatient follow-up.

The criteria for inpatient admission vary across insurers. Medicare defines the inpatient level of care based on medical necessity, condition severity, and an expected hospital stay spanning at least two midnights, with certain exceptions^17^. Observation status, as an outpatient classification, has lower hospital reimbursement rates and higher out-of-pocket costs for patients. Under Medicare, observation stays are covered under Part B rather than Part A, meaning patients without Part B coverage would be responsible for the hospital stay out of pocket^17^. High observation rates negatively impact hospital throughput, particularly in hospitals with high occupancy. By shifting certain hospital-based outpatient services, such as observation admissions, to clinic-based outpatient care, hospitals can improve bed availability for higher-acuity patients while also benefiting financially. Inpatient care generally has a significantly higher reimbursement rate than outpatient observation stays, making efficient patient placement a key strategy for hospital operations.

Upstate ranked 7 th of 160 New York State hospitals for most minutes spent by patients in a hospital’s emergency department in 2021–2022.^18^ As the COVID pandemic entered an endemic phase, Upstate experienced a staffing crisis, resulting in long emergency department wait times, limited bed availability, and reduced ability to accept transfers for medically necessary specialty services and time-sensitive surgeries. To overcome the constrained resources, innovative strategies were developed to optimally utilize staffing resources, increase capacity, and improve the quality of care for patients.

This paper describes how the Upstate Hospital Medicine Division in the Department of Medicine has removed ED discharge friction points using a novel approach to reduce low-acuity admissions by streamlining ambulatory evaluations for low-acuity conditions presenting to the ED, such as low-risk chest pain. Significant barriers to timely discharge included lack of access to post-acute ambulatory care and the retention of patients in an observation status to complete time-sensitive, but not urgent or emergent, testing. While this manuscript highlights one example of how the model improved inpatient capacity, it is designed with the potential to expand to offer efficiencies in additional clinical conditions.

The Upstate Connect Care Project (CCP) was designed to triage appropriate ED patients to a dedicated clinic, the Connect Care (CC) clinic, that would provide post-discharge follow-up, testing, and some therapeutic procedures for patients. The CC clinic was also designed to serve as a bridge to a permanent primary care practitioner for those who lack such a relationship. The CC clinic provides the health system with a mechanism for diverting patients with ambulatory-responsive conditions from being admitted under observation to a lower acuity ambulatory setting. In addition to improving capacity and throughput, the CC clinic was designed to offer a streamlined, lower cost option for patients that is also fiscally sustainable for the hospital. The Upstate CC clinic is an example of one of the novel care models piloted at SUNY Upstate University Hospital to address challenges of access, capacity, outcomes, and high costs by shifting care from inpatient to an efficient ambulatory environment.

### Program Description

**Figure 1:**
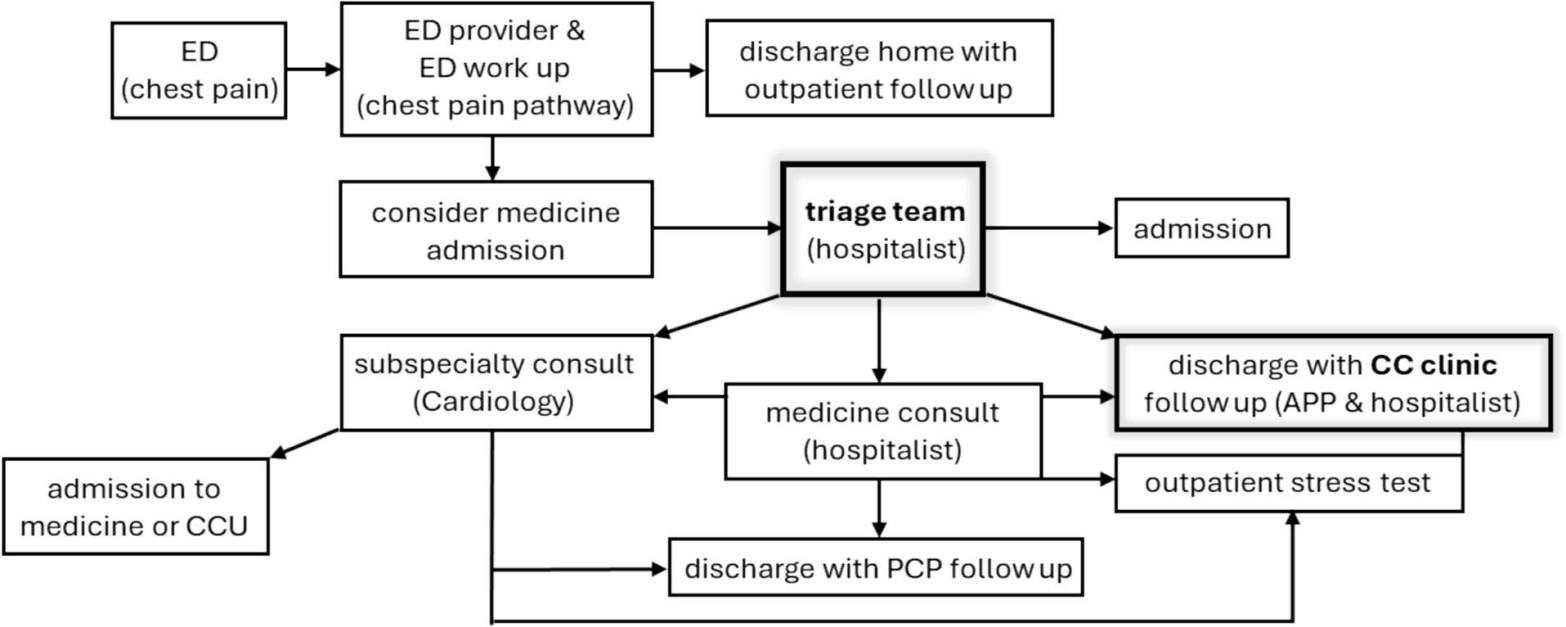
Workflow of admission at Upstate. Both ED providers and hospitalists (triage team and medicine consult) work together to determine the safe disposition plan for patients presenting in the ED needing further evaluation. The Chest Pain Pathway is shown as an example that uses initial ED work-up (high-sensitivity troponin, EKG) and the patient’s history and risk factors (HEART score). Patients are either admitted for further testing/intervention or discharged with outpatient follow-up with PCP or at CC clinic with or without a stress test.

**Figure 2:**
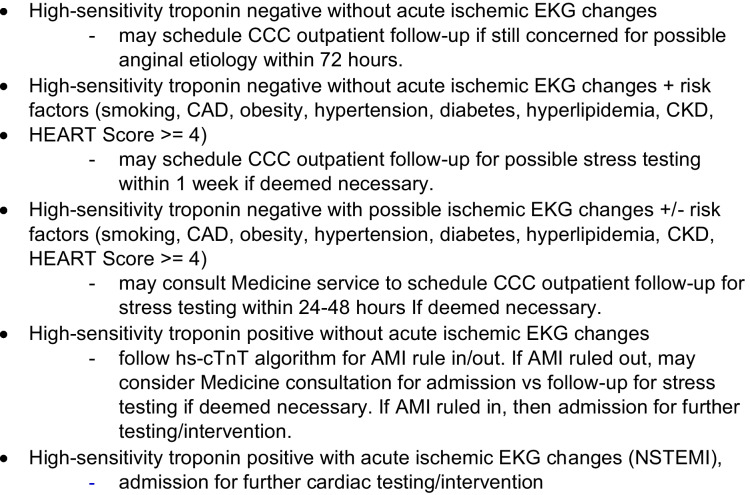
Chest Pain Pathway. Developed in collaboration between Hospital Medicine and ED Department. Patients are triaged based on EKG, high-sensitivity troponin, and HEART score.

The Upstate CCP consists of two components, a triage team and the CC clinic. The Connect Care Project aims to transition certain hospital-based outpatient services, including observation-level care, to clinic-based care by expediting diagnostic workups at the CC clinic and facilitating timely access to tests such as stress tests, CT scans, and MRIs. The triage team collaborates with the emergency department (ED) to identify patients whose clinical needs can be managed in the CC clinic. The triage team manages requests for admissions from the ED, outpatient clinics, and transfers from other hospitals. By coordinating with case managers and utilization management, the triage team ensures safe and efficient admissions and transfers by connecting appropriate providers with bed management. For patients whom medical care could be shifted from hospital based to clinic based, the triage team arranges for additional assessment of the patient, which includes a medicine consult team evaluation in the ED, followed by outpatient evaluation and treatment in the CC clinic, in another ambulatory subspecialty clinic, or with the patient’s primary care physician (Fig. 1). The CC clinic offers procedures, including paracentesis, thoracentesis, and vascular access placement, as well as accelerated condition-specific ambulatory clinical care pathways. Same-day appointments are available at the CC clinic for those who need prompt follow-up evaluations after ED or hospital discharge. For patients who do not have a primary care physician (PCP), the CC clinic will bridge the gap in care by offering outpatient visits while working with patients to establish care with a new PCP. Examples of clinical pathways developed for outpatient evaluation and treatment of common conditions include low-risk chest pain (Fig. 2), low-risk syncope, and uncomplicated cellulitis. For patients presenting with chest pain, the triage team identifies those who are eligible for same-day stress testing while in the ED without an admission and those who can be safely followed with an outpatient stress test within 48 hours of the initial ED visit. For these patients, the CC clinic has collaborated with the hospital-based stress test center to accommodate patients who would benefit from a stress test promptly. In collaboration with the hospital finance office, the CC clinic can schedule outpatient stress tests regardless of the insurance status. There is a dedicated administrative staff to handle prior authorization if needed for those undergoing a stress testing at the CC clinic.

The CC clinic is a multi-disciplinary initiative that focuses on improving throughput, patient care continuum, and safe transition of patients from both ED and inpatient care to outpatient settings. This ambulatory clinic is physically linked to the Upstate University Hospital and is staffed by Advanced Practice Providers (APPs), nurses, and administrative staff, all under the supervision of a dedicated hospitalist. There is a dedicated 24-hour scheduling call line to streamline appropriate follow-up with a reduced interval between discharge and outpatient follow-up. The clinic is equipped to provide services such as lab work, X-rays, CT scans, and stress tests within 48 hours of ED and hospital discharge. This study focuses on observation hospital stays, particularly for chest pain rule-out cases, as chest pain is a common reason for observation status, and because of the structured care pathway within CCP that enables stress testing within 48 hours of hospital presentation, allowing for safe outpatient timely evaluation of chest pain at the CC clinic.

#### Participants

Patients admitted to observation status between 03/2020 and 04/2023 were examined. To control for the impact of the COVID-19 pandemic, data were retrospectively collated from 03/2020 to coincide with the timing of the first COVID-19 patients observed in the region surrounding the host site. The pre-intervention period includes 18 months from 3/2020 until 10/2021, and the post-intervention period was coded beginning in 11/2021 and for 20 months after the implementation of the CCP (the intervention).

Chest pain is one of the most common medical conditions admitted for further workup under observation. Patients presenting with chest pain were risk stratified using a chest pain pathway created collaboratively between the Department of Emergency Medicine and the Division of Hospital Medicine that considered high-sensitivity troponin, EKG, and the HEART score in clinical context (Fig. 2). Patients who did not require inpatient admission were either discharged home to follow-up with the primary care physician, received a stress test in the ED, were discharged with a close follow-up with the Connect Care for a stress test within 48 h, or were admitted to the hospital under observation. This study was granted an exemption from review by the Upstate institutional review board (IRB).

#### Data Analysis

The primary outcomes assessed were the mean numbers of admissions to the medicine service under observation before and after the implementation of the CCP. We analyzed observation admissions to the institution’s Medicine service, both total and chest pain specific, and compared them to all observation admissions across other services excluding Medicine.

Upstate operates two full-service emergency rooms, both of which received the CCP intervention and which receive cases from the same city and region. *A priori* testing of patient volume revealed no differences between the two emergency rooms, so they were treated together as one facility for the purposes of this study. The analysis was then conducted in two phases: descriptive and regression analyses.

#### Descriptive Analysis

In the first phase, we calculated the mean monthly number of ED encounters and observation admissions before and after CCP implementation. This included:

- Total number of ED encounters across the system
- All ED encounters for chest pain
- Chest pain admissions under observation
- All admissions to Medicine under observation
- Total admissions under observation from all services except Medicine

Independent sample *T*-tests were conducted to compare the mean monthly numbers across the pre-intervention period vs. the post-intervention period for each of the encounter and admission outcomes.

#### Regression Analysis

Next, we examined mean monthly admissions under observation before and after the CCP intervention, adjusting for the overall patient volume using ED encounters and ED encounters for chest pain as covariates. Although count data was used, we employed both a simple and covariate-controlled ordinary least squares (OLS) linear regression procedure for simplicity and clarity in reporting. Two models were run for each of the five admission outcomes:

1. Admission = Constant + β1 (Pre/Post)
2. Admission = Constant + β1 (Pre/Post) + β2 (Total Encounters) + β2 (Chest Pain Encounters)

Since count data was used, we validated the OLS regression analyses with Poisson linear procedures, reporting the exponentiated *β* coefficients for each admission outcome, adjusting for the same covariates as in the linear regressions.

Standard diagnostic tests were calculated alongside the regression analyses, including assessment of collinearity between covariates, and for autocorrelation in the time series data. All statistical procedures were performed in SPSS v. 29.

### Program Evaluation

A total of 305,207 cases were presented to the Upstate Emergency Department over the entire study period. A breakdown of case totals is shown in Table 1.

**Table 1:** Tallies of Total ED Encounters, Chest Pain ED Encounter, and Admissions Under Observations, via *t*-Test, Between Pre- and Post-Intervention (March 2020–April 2023)

Period	Pre-period (3/20–9/21)	Post-period (10/21–4/23)	Grand total
*Total ED cases*	151,954	153,253	305,207
*Total chest pain cases presented to the ED*	5701	5831	11,532
*Total chest pain admissions under observation status*	1039	334	1373
*Total admissions to medicine under observation*	5188	2937	8125
*Total admissions under observation from all services except medicine*	959	861	1820

The mean number of total ED encounters per month was 7997.58 between March 2020 and October 2021, and 8065.95 ED encounters between November 2021 and April 2023. The mean number of chest pain-related ED encounters per month increased from 300.05 in the pre-period to 306.89 in the post-period. Neither of the pre/post differences in ED volume, however, was statistically significant. The mean number of all admissions to medicine under observation per month and chest pain admissions under observation per month significantly decreased from the pre- to post-period. In contrast, the mean number of observation admissions per month to all services (excluding medicine) did not significantly change, as shown in Table 2.

**Table 2:** Comparison of Means via *t*-Test Between Pre- and Post-Intervention (March 2020–April 2023)

Duration from 03/2020 to 04/2023	Pre Post	Mean	SD	*p*
*Total ED encounters per month*	0	7997.58	1290.01	NS
	1	8065.95	651.31	
*ED encounters of chest pain per month*	0	300.05	37.42	NS
	1	306.89	34.43	
*Chest pain admissions under observation per month*	0	54.68	14.26	<0.001
	1	17.58	5.06	
*All admissions to medicine under observation per month*	0	273.05	57.38	<0.001
	1	154.58	24.10	
*Total admissions under observation per month from all services except medicine*	0	50.47	11.68	NS
	1	45.32	8.30	

Chest pain admissions under observation decreased significantly in the post-period, with an average reduction of about 37 observation admissions per month following the implementation of the CCP (*β*= −37.105, *p*<0.001). This effect remained robust even after adjusting for overall and chest-pain-specific ED volume (*β*= −37.794, *p*<0.001). A similar decline was observed in the mean number of all admissions to medicine under observation per month, as described further in Table 3. The OLS linear regressions were validated with parallel results obtained via Poisson regression (Table 3).

**Table 3:** OLS Regression of Monthly Observation Admissions, Pre vs Post, (1) Unadjusted and (2) Adjusted for Total ED Encounters and ED Encounters of Chest Pain Between March 2020 and April 2023. Exponentiated *β*, Representing Post-intervention, from Poisson Regression Is Included as well

	Linear unadjusted		Linear adjusted		Poisson	
	*β*	*p*	*β*	*p*	Exp β	*p*
*Chest pain admissions under observation*	−37.105	<.001	−37.794	<0.001	0.321	<0.001
*All admissions to medicine under observation*	−118.474	<.001	−119.657	<0.001	0.565	<0.001
*Total admissions under observation from all services except medicine*	−5.158	NS	−5.331	NS	0.895	<0.001

## DISCUSSION

This paper highlights the outcomes of a hospital-initiated project to address the growing demand for access and capacity in a healthcare system operating at 100% capacity utilization. The intervention aimed to create an ambulatory alternative to hospital-based outpatient evaluation (observation) for appropriate patients, thereby reducing reliance on inpatient resources. While this paper focuses on a specific outcome of the intervention, we anticipate additional benefits related to hospital capacity, including a reduction in LOS, a key indicator of hospital efficiency and patient care.^19^ The CCP, developed by the Division of Hospital Medicine at SUNY Upstate Medical University, provides streamlined access to post-acute care follow-up for patients discharged after inpatient or ED encounters. This model enhances hospital capacity and throughput, while providing cost-effective and a smooth transition from inpatient to outpatient care.

Our project was primarily implemented in the Division of Hospital Medicine, focusing on hospital admissions under observation. Chest pain admissions under observation were selected as an example for this paper, as chest pain is one of the most frequently admitted conditions in this category. Our results demonstrate a significant decrease in both total hospital admissions under observation in the medicine department and chest pain admissions under observation following CCP implementation, despite stable total ED volume and chest pain-related ED visits (Table 2). Conversely, observation admissions across all services (excluding medicine), where our project was not implemented, did not show any significant change. Throughout the study period, the overall hospital capacity utilization remained constant. By reducing the number of observation admissions to the medicine service, the CCP freed an average of 214 bed days per month, increasing bed availability for patients with acute medical conditions. This change resulted in an estimated positive financial impact of $389,536 monthly for our hospital system. The calculation was based on the difference in reimbursement rates between inpatient and observation levels of care at SUNY Upstate Hospital, using the average difference across all payers with an average LOS of 5 days for inpatient and 2 days for observation.

One of the major limitations of the study is that possible negative outcomes experienced by patients discharged with CC clinic follow-up, including return to the ED within 48 h of discharge or readmission within 72 hours, remain unknown at this time and are currently under study. Preliminary data from patients seen at their follow-up CC clinic appointment suggest no significant adverse events; however, a more detailed study has not yet been completed. Further investigation of patient outcomes following the implementation of the CC clinic would also allow a more comprehensive assessment of the impact of the CCP on patient care and would identify areas of opportunity to improve the quality of transition from acute to post-acute care. Another area for further investigation is the impact of transitioning hospital-based outpatient care to a clinic-based setting on patient experience. We are currently working with the patient experience committee and the patient relations team to better understand how the CC care has influenced patients from the patient’s perspective as well.

A significant challenge in hospital operations is the admission of patients with conditions that could be managed in an outpatient setting, primarily due to limited resources or the inability to ensure timely follow-up. Many of these cases involve urgent outpatient infusions and blood transfusions that cannot be accommodated promptly by existing outpatient clinics or emergency departments (ED). Similarly, patients discharged from the hospital or presenting with urgent outpatient needs often lack timely access to follow-up care, leading to unnecessary hospital utilization. The CC clinic has proven to be an effective solution for transitioning certain hospital-based outpatient services to a clinic setting, thereby increasing hospital capacity to accommodate patients requiring inpatient care.

To further enhance hospital throughput and optimize patient care, we aim to expand the CC clinic to encompass three key areas: infusion services, timely follow-up for hospital and ED discharges, and urgent walk-in clinic. The CC clinic is equipped to provide urgent infusions referred by outpatient clinics and blood transfusions for patients discharged from the hospital. Patients who are discharged from the hospital or ED will also be able to receive appropriate follow-up care to prevent readmissions. The CC clinic will also expand to offer outpatient visits for patients with urgent healthcare needs who cannot be accommodated by their primary care provider, reducing unnecessary hospital or ED visits. By expanding the scope of the Connect Care Clinic, we can improve patient care, optimize hospital resources, and enhance overall healthcare efficiency. The findings of this project can be applied to different hospital systems of varying acuity by identifying the post-acute care needs for the most common medical conditions for that institution and arranging for streamlined follow-up within the hospital system’s ambulatory clinics.^20^ Our approach will allow us to establish additional interdisciplinary projects between the Division of Hospital Medicine and the Department of Emergency Medicine to bridge the gaps between inpatient and outpatient care.

## Data Availability

The data that support the findings of this study are available from the corresponding author upon request.

## Declarations

We confirm that all authors have approved the manuscript and agreed to its submission.
